# Dataset for file fragment classification of image file formats

**DOI:** 10.1186/s13104-019-4812-0

**Published:** 2019-11-27

**Authors:** Reyhane Fakouri, Mehdi Teimouri

**Affiliations:** Information Theory and Coding Laboratory, University of Tehran, Tehran, Iran

**Keywords:** Classification, File formats, File fragments, Image file formats

## Abstract

**Objectives:**

File fragment classification of image file formats is a topic of interest in network forensics. There are a few publicly available datasets of files with image formats. Therewith, there is no public dataset for file fragments of image file formats. So, a big research challenge in file fragment classification of image file formats is to compare the performance of the developed methods over the same datasets.

**Data description:**

In this study, we present a dataset that contains file fragments of ten image file formats: Bitmap, Better Portable Graphics, Free Lossless Image Format, Graphics Interchange Format, Joint Photographic Experts Group, Joint Photographic Experts Group 2000, Joint Photographic Experts Group Extended Range, Portable Network Graphic, Tagged Image File Format, and Web Picture. Corresponding to each format, the dataset contains the file fragments of image files with different compression settings. For each pair of file format and compression setting, 800 file fragments are provided. Totally, the dataset contains 25,600 file fragments.

## Objective

A large amount of Internet traffic is used for exchanging image file formats. As the sizes of these files are usually much bigger than the maximum network packet size, the files are segmented into fragments. The fragments generated by various users are transmitted over the network. Some of these fragments can be received by the network surveillance unit. The network surveillance unit may wish to detect the file format of each fragment for network forensics purposes.

Some researches have been carried in the field of file fragment classification of image file formats [1, 2]. There are a few publicly available datasets of files with different formats [3]. Therewith, there is no public dataset for file fragments of image file formats. This makes it difficult for other researchers to compare the proposed methods with the existing methods.

In this study, we present a dataset that contains file fragments of ten image file formats: Bitmap (BMP), Better Portable Graphics (BPG), Free Lossless Image Format (FLIF), Graphics Interchange Format (GIF), Joint Photographic Experts GROUP (JPEG), Joint Photographic Experts Group 2000 (JPEG 2000), Joint Photographic Experts Group Extended Range (JPEG XR), Portable Network Graphic (PNG), Tagged Image File Format (TIFF), and Web Picture (WEBP). Corresponding to each format, the dataset contains the file fragments of image files with different compression settings.

## Data description

First, the whole set of raw image files is downloaded from the RAISE project [4]. These raw files are then converted in order to obtain image files in ten different formats: BMP, BPG, FLIF, GIF, JPEG, JPEG 2000, JPEG XR, PNG, TIFF, and WEBP. For each image file format, different compression settings are considered. Each raw image is converted into a specific file format using a particular compression setting. So, the contents of any two image files are not the same.

32 pairs of file format and compression setting are considered. For each pair of file format and compression setting, we have 160 compressed images. So, totally we have 5120 image files. Each of these files is segmented into 1 Kbyte (i.e. 1024 bytes) fragments. Then, five fragments are randomly selected among the fragments of each file. Before randomly selecting the fragments, 12.5% of the initial fragments and 12.5% of the final fragments of each file are discarded. This is to ensure that the fragments do not contain the file headers or trailers.

For each pair of file format and compression setting, we have 800 file fragments. So, the dataset of file fragments contains 25,600 file fragments. The dataset is partitioned according to 32 different pairs of file format and compression setting. Each partition is represented by an individual data set shown in Table 1. For example, data set 1 (i.e. BMP1.dat) contains 800 fragments of uncompressed BMP files. Data sets are provided in a generic binary data file format with .dat file extension.

**Table 1:** Overview of data files/data sets

Label	Name of data file/data set	File types (file extension)	Data repository (DOI)
Data file 1	SettingsTable	Portable document format (.pdf)	OSF (10.17605/OSF.IO/YH3XP)
Data file 2	ConversionSettings	Archive file format (.zip)	OSF (10.17605/OSF.IO/YH3XP)
Data file 3	ReadFragments	Matlab script file (.m)	OSF (10.17605/OSF.IO/YH3XP)
Data set 1	BMP1	Generic binary data (.dat)	OSF (10.17605/OSF.IO/YH3XP)
Data set 2	BMP2	Generic binary data (.dat)	OSF (10.17605/OSF.IO/YH3XP)
Data set 3	BMP3	Generic binary data (.dat)	OSF (10.17605/OSF.IO/YH3XP)
Data set 4	BPG1	Generic binary data (.dat)	OSF (10.17605/OSF.IO/YH3XP)
Data set 5	BPG2	Generic binary data (.dat)	OSF (10.17605/OSF.IO/YH3XP)
Data set 6	BPG3	Generic binary data (.dat)	OSF (10.17605/OSF.IO/YH3XP)
Data set 7	BPG4	Generic binary data (.dat)	OSF (10.17605/OSF.IO/YH3XP)
Data set 8	FLIF	Generic binary data (.dat)	OSF (10.17605/OSF.IO/YH3XP)
Data set 9	GIF1	Generic binary data (.dat)	OSF (10.17605/OSF.IO/YH3XP)
Data set 10	GIF2	Generic binary data (.dat)	OSF (10.17605/OSF.IO/YH3XP)
Data set 11	JPEG1	Generic binary data (.dat)	OSF (10.17605/OSF.IO/YH3XP)
Data set 12	JPEG2	Generic binary data (.dat)	OSF (10.17605/OSF.IO/YH3XP)
Data set 13	JPEG3	Generic binary data (.dat)	OSF (10.17605/OSF.IO/YH3XP)
Data set 14	JPEG4	Generic binary data (.dat)	OSF (10.17605/OSF.IO/YH3XP)
Data set 15	JPF1	Generic binary data (.dat)	OSF (10.17605/OSF.IO/YH3XP)
Data set 16	JPF2	Generic binary data (.dat)	OSF (10.17605/OSF.IO/YH3XP)
Data set 17	JPF3	Generic binary data (.dat)	OSF (10.17605/OSF.IO/YH3XP)
Data set 18	JPF4	Generic binary data (.dat)	OSF (10.17605/OSF.IO/YH3XP)
Data set 19	JXR1	Generic binary data (.dat)	OSF (10.17605/OSF.IO/YH3XP)
Data set 20	JXR2	Generic binary data (.dat)	OSF (10.17605/OSF.IO/YH3XP)
Data set 21	JXR3	Generic binary data (.dat)	OSF (10.17605/OSF.IO/YH3XP)
Data set 22	JXR4	Generic binary data (.dat)	OSF (10.17605/OSF.IO/YH3XP)
Data set 23	PNG1	Generic binary data (.dat)	OSF (10.17605/OSF.IO/YH3XP)
Data set 24	PNG2	Generic binary data (.dat)	OSF (10.17605/OSF.IO/YH3XP)
Data set 25	PNG3	Generic binary data (.dat)	OSF (10.17605/OSF.IO/YH3XP)
Data set 26	PNG4	Generic binary data (.dat)	OSF (10.17605/OSF.IO/YH3XP)
Data set 27	TIF1	Generic binary data (.dat)	OSF (10.17605/OSF.IO/YH3XP)
Data set 28	TIF2	Generic binary data (.dat)	OSF (10.17605/OSF.IO/YH3XP)
Data set 29	WEBP1	Generic binary data (.dat)	OSF (10.17605/OSF.IO/YH3XP)
Data set 30	WEBP2	Generic binary data (.dat)	OSF (10.17605/OSF.IO/YH3XP)
Data set 31	WEBP3	Generic binary data (.dat)	OSF (10.17605/OSF.IO/YH3XP)
Data set 32	WEBP4	Generic binary data (.dat)	OSF (10.17605/OSF.IO/YH3XP)

Data file 1 (i.e. SettingsTable.pdf) contains a table that specifies 32 pairs of file format and compression setting. In this table, the software program employed for generating each file format is also specified. Data file 2 (i.e. ConversionSettings.zip) contains several screenshots of the software programs that display the employed compression settings. Data file 3 (i.e. ReadFragments.m) is a script in MATLAB language that reads all the fragments from one or more specific data sets. By running this script and selecting some data set files, the fragments contained in these data sets are read and stored in a variable name Dataset. Variable Dataset is a MATLAB cell array with two rows. Each column in this cell array corresponds to one of the selected data sets. The first element of each column is a string value that specifies the data set file name. The second element of each column is a structure array with only one field named fragments. Dataset{2,i}(j).fragments (j = 1,2,…,160) is a cell array with length 5 that contains five fragments of the jth file in the selected data set i.

## Limitations

- The size of the fragments is considered to be fixed and equal to 1024 bytes.
- A defined subset of file formats and compression settings are considered.

## Data Availability

The data described in this Data note can be freely and openly accessed on OSF at 10.17605/OSF.IO/YH3XP [5]. Please see Table 1 and reference list for details and links to the data.

## Abbreviations

BMP: Bitmap
BPG: Better Portable Graphics
FLIF: Free Lossless Image Format
GIF: Graphics Interchange Format
JPEG: Joint Photographic Experts Group
JPEG 2000: Joint Photographic Experts Group 2000
JPEG XR: Joint Photographic Experts Group Extended range
PNG: Portable Network Graphic
TIFF: Tagged Image File Format
WEBP: Web Picture
